# Death from an Acute Attack of Gout

**Published:** 1854-11

**Authors:** Caspar Morris

**Affiliations:** Consulting Physician to the Philadelphia Hospital


### MEDICAL EXAMINER.

NEW SERIES.—NO. CXIX.—NOVEMBER, 1854.

### ORIGINAL COMMUNICATIONS.

*Death from an acute attack of Gout.* By CASPAR MORRIS, M. D., Consulting Physician to the Philadelphia Hospital.

Death from an acute attack of gout is, I believe, a rare event; at least, such is the result to which an experience of thirty years, during which I have certainly seen many severe cases, both of the regular and irregular forms of the disease, has brought me. The case I am about to narrate, is the only one which has ever terminated fatally under my observation; organic disease of the heart or kidneys, giving rise to effusion in the cavities, being the more common mode in which gout produces death.

The patient was a gentleman of about fifty-five years of age, who had inherited the gouty diathesis; and in the early years of manhood had lived freely, but during the last twenty years had never or very rarely tasted wine, malt liquors or any other alcoholic drink. His business was one which gave him moderate exercise, but subjected him to great anxiety. His habits of life were regular, and he ate largely of animal food. Twice in each year, he suffered from severe podagra, generally affecting both feet, but yielding to the use of colchicum and free purgation. About three years before his death, he had a violent attack of gout in the bowels, evidenced by the most agonizing pain, obstinate constipation and great vascular excitement, with heat of skin and coated tongue; blood-letting largely practised, and opium, afforded entire relief.

In the month of August, 18—, he went to his counting-house in usual health; but as he sat reading the newspaper, his son observed him thrusting the paper strongly to one side, while his head was obstinately deflected in the opposite direction, and on speaking to him found his intellect dull, and his tongue not under control; there was no further evidence of paralysis. I found his pulse nearly normal; and a few ounces of blood drawn by cups from the nape of the neck, followed by a brisk mercurial cathartic, sufficed to restore the regular balance of his system. About four weeks after this, I removed a large steatomatous tumor from the scalp. The entire wound, which was quite extensive, healed strictly by the first intention; there was not the slightest suppuration. I mention these facts merely to exhibit the state of his system.

On the morning of the 20th of September, I was called to see him at an early hour. He had passed a restless night and been chilly, and had voided an inordinate quantity of urine, filling entirely one vessel of the largest size, and nearly another; I should think the quantity was not less than three or four quarts. I found him with a very hot and dry skin, a vigorous pulse, tongue covered with the dense, white coat, which I have commonly found in gouty patients, respiration natural, and head aching severely. Influenza was prevailing at the time in the city.

I prescribed for him an active cathartic of calomel, followed by castor oil. At my evening visit, I found that though the purgative had acted freely, the febrile condition was exasperated. The pulse was very tense and full, like that produced by a hypertrophied heart, the head-ache was greater, the tongue more densely loaded, and he had a sense of gastric oppression, with much uneasiness in the throat. There was no evidence of disease in the mucous membrane; it was rather dysphagia. I directed the loss of twelve ounces of blood from the arm, a mustard pediluvium, and a dose of Dover's powders. At my visit next morning, I found he had slept and perspired freely under the influence of the diaphoretic and anodyne; but the sensation of distress in the throat had increased, and there was difficulty of respiration of a very peculiar character. There was no stitch in the side, nor acute pain, nor soreness as in pleurisy or pleuro-pneumonia; but every attempt at inspiration was met by a strong expiratory action, as though caused by a spasm of the diaphragm. It was now that the idea of gout presented itself, and in order to meet this view of the case, I administered wine of colchicum in free doses, frequently repeated; and directed twelve ounces of blood to be drawn by cupping glasses applied between the scapulæ. That drawn from the arm on the previous day, was found to have coagulated very firmly, having a concave surface with a dense fibrinous coat upon it. Warm cataplasms were applied to the throat. In the course of the day, there was some settled pain in the right side, and his pulse being still firm and active, ten ounces more of blood were taken from the arm, by which the force of the pulse was very decidedly reduced. A blistering plaster was now applied over the seat of pain, and pills of mass hydrarg. and Dover's powder were added to the treatment. A violent spasmodic cough, accompanied by a very sharp pain in the left side, induced me to apply cupping glasses over the seat of pain, without drawing any blood, and when they had produced all the irritation possible, a blistering plaster was placed over the spot. This drew well, and the night was passed with tolerable comfort under the influence of the Dover's powder.

A dose of calcined magnesia was given in the morning, which operated freely, and as the pulse had lost its frequency and tension, and the pain was removed, chicken broth was given for nourishment, and the colchicum and mass hydrarg. were withheld. The skin continued moist during the day, and the respiration easy. Dover's powder was given at night in order to secure rest, which was accomplished, and the morning found him apparently convalescent. At mid-day I found he had been much exhausted by two very copious alvine discharges, and in order to prevent a recurrence of them, I gave two drachms of paregoric. In about two hours after taking this, he was seized with a violent fit of spasmodic cough, which lasted half an hour; and on my visit in the evening, I found his respiration very hurried, and a recurrence of the pain in the side, while his bowels were distended with gas. Warm cataplasms, a stimulating foot bath, and the

Dover's powders, and blue mass were resorted to promptly, but without affording any relief. The respiration became more and more hurried and difficult, the pulse very feeble, and the skin cool. Percussion proved that there was neither condensation of the lung, nor effusion into the pleural sac, to cause the distress, which amounted to orthopnœa; neither was there any friction sound. Fully satisfied of the correctness of the view I had taken of the case, and that it was one of gout affecting the diaphragm, I again resorted to mustard pediluvia and cataplasms, and gave hot wine whey and carbonate of ammonia, in large and frequently repeated doses. Though the pulse became very feeble, his general muscular force appeared unabated, so that he persisted in rising to the chair to effect the discharge of bowels and bladder, and turned and raised himself in the bed freely without aid. Dr. Pepper now saw him with me, taking a similar view of the case. The use of colchicum was resumed and morphia given, and as his stomach gave way, and he had a sense of nausea, Tinct. Opii 60 drops was given by injection into the rectum, but without affording any relief. At my next visit, which was within two hours, I found the belly tympanitic, and the restlessness and difficulty of breathing much augmented, while the pain had fixed itself in the right crus of the diaphagram. Mustard cataplasms were freely applied, and an attempt to solicit the discharge of the flatus, was made by the administration of a large enema of spirits of turpentine in an infusion of assafœtida. A tube was also passed into the bowel, in the hope that by relieving the spasmodic contraction of the sphincter ani, the bowels would expel their contents. The muscular coat of the intestines had lost its power of contraction. He even rose and sat upon the stool, but without succeeding in evacuating the bowels. We therefore placed him on the free use of calomel and opium, and directed the application of camphorated chloroform on flannels, covered with oiled silk, to the seat of pain. At our visit in the morning, we found that he had slept a few minutes at a time frequently during the night, but was quite wakeful and conscious at the time of our visit, though he had taken five grains of opium during the twelve hours. There was no abatement of the pain; the orthopnœa was so great that he could not lie down a moment. The respiration was exceedingly hurried, accompanied by constant moans. He had singultus with the ejection from the stomach of large quantities of matter, looking like the black vomit of yellow fever, which coated his tongue and dried upon the surface; his pulse was exceedingly feeble, and in a few hours he expired.

The autopsy showed the stomach and bowels loaded with matter like that ejected from the mouth, and distended by gas, but no trace of any organic lesion. The mucous coat of the stomach was healthy, as were also the heart, lungs and pleura, liver and kidneys.

A specimen of the matter ejected from the stomach was subjected to the microscope by Professor Liedy, who reports, "I saw no blood corpurcles. It consisted of liquid, brownish granules, oil globules, phytoid filaments, probably derived from the mouth and fauces, vibrios, a few starch granules, recalling the sarcina, but most probably fragments of vegetable food."

